# Data Resource: Children and Family Court Advisory and Support Service (Cafcass) public family law administrative records in England

**DOI:** 10.23889/ijpds.v5i1.1159

**Published:** 2020-03-26

**Authors:** SJ Bedston, RJ Pearson, MA Jay, K Broadhurst, R Gilbert, L Wijlaars

**Affiliations:** 1 Centre for Child and Family Justice Research, Department of Sociology, Lancaster University, LA1 4YT; 2 Legal Epidemiology Group, Population, Policy and Practice Research and Teaching Department, UCL Great Ormond Street Institute of Child Health, 30 Guilford Street, London, WC1N 1EH; 3 NIHR Children’s Policy Research Unit, Population, Policy and Practice Research and Teaching Department, UCL Great Ormond Street Institute of Child Health, 30 Guilford Street, London, WC1N 1EH

## Abstract

**Introduction:**

In England, in cases of child maltreatment or neglect, the state can intervene through the family court to remove children from their family home and place them in out-of-home care. The Children and Family Court Advisory and Support Service (Cafcass) collects and maintains administrative records of all public family law cases in England. While these national records are primarily used to monitor and manage the workflow of Cafcass teams across England, researchers have re-purposed this data for analysis to understand the drivers and outcomes of public family law intervention.

**Data contents:**

The administrative dataset is a reflection of the cases Cafcass is involved with and the extent of that involvement. The dataset contains information about the local authority that makes an application to initiate public family law proceedings, the children and families involved, and the duration and details of the case. Between 1 April 2007 and 31 March 2019, Cafcass captured information on approximately 172,100 public family law cases, involving 282,300 children, and 349,600 adults (of which 289,300 are recorded as biological parents). Amongst the information recorded are the relations between adults and children, making it possible for researchers to identify family groups. Additionally, recording practices at Cafcass have improved over time, this has increased the availability of demographic information of all those involved, as well as child’s final legal outcome.

**Data access:**

Researchers can apply to the Secure Anonymised Information Linkage databank (SAIL) for access to the Cafcass pseudonymised administrative data extract, where it is refreshed bi-annually.

**Keywords:**

children, out of home care, family relations, family law

## Introduction

In cases of child maltreatment or neglect, the state can intervene through the family court to remove children from the family home and place them in out-of-home care (OOHC). Although the group of children experiencing OOHC is relatively small (3.3% by age 18 year in England [1]), removal from parental care is one of the most intrusive interventions children and their families can experience.

Despite this, little research evidence is available on the effects of OOHC on children, their siblings or their parents. A range of disciplines, such as the nascent field of legal epidemiology, which investigates health and social outcomes of law and legal processes, require high quality data with sufficiently large, representative samples or population coverage [2]. The Department for Education (DfE) collects limited data on looked-after children in the Children Looked After (CLA) return which is available to researchers [3]. However, this national data collection does not allow for identifying siblings and parents, or the examination of family court processes, nor does it capture those who go to court and do not enter local authority care. In this data resource profile, we present a dataset curated by the Children and Family Court Advisory and Support Service (Cafcass), which, as part of its routine service delivery and planning operations, records data on public and private family court proceedings in England involving children which includes information on family structure.

**Figure 1: A summary of how the three different national datasets capture children who come into initial contact with children’s services.**
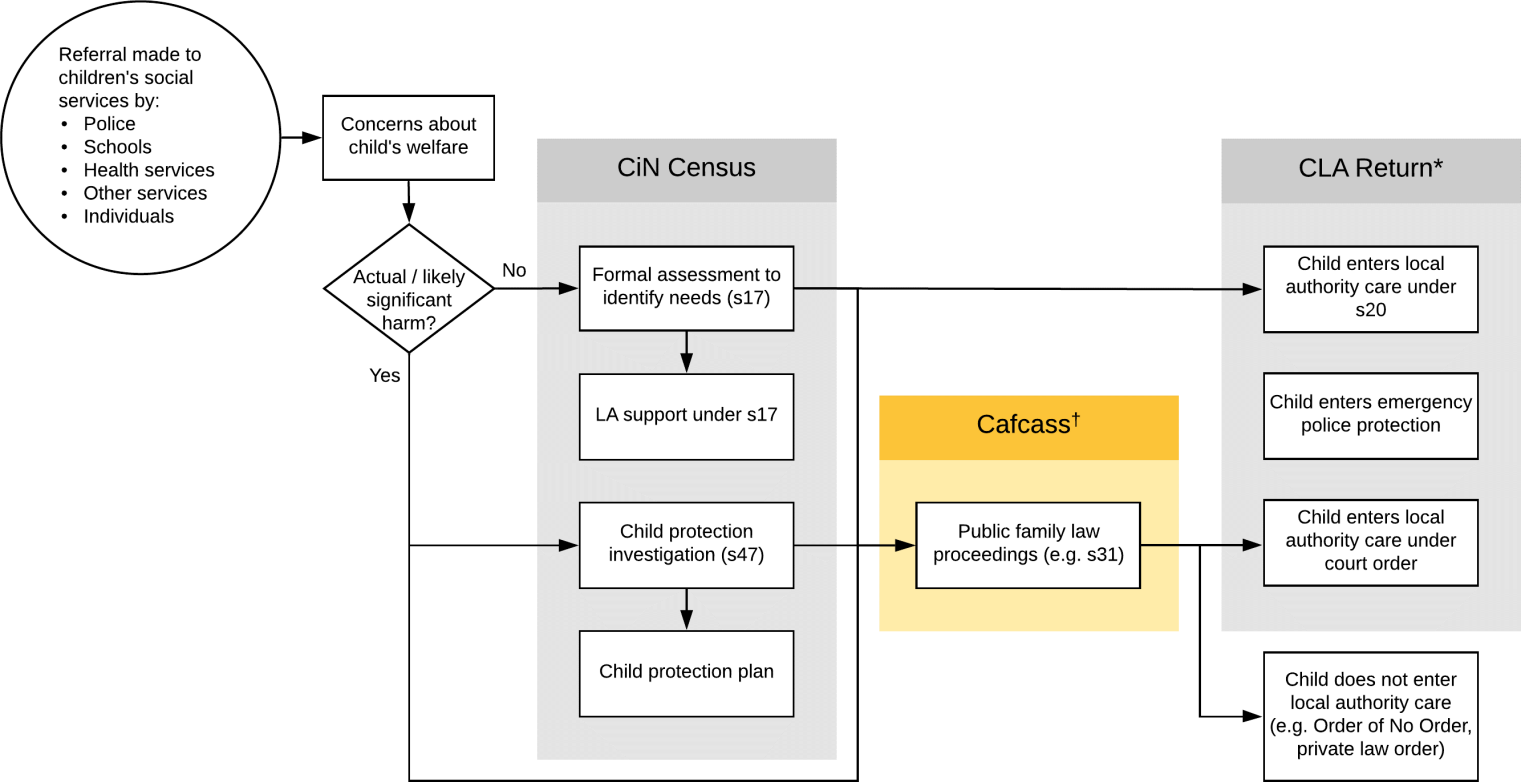
*All children in the care of a local authority are, by definition, also children in need (s17 Children Act 1989). Therefore, these children are in both the CiN Census and CLA return.

†The Cafcass data contains information on all family members relevant to the case, not just children.

Local authorities have duties and powers under the Children Act 1989 to safeguard and promote the welfare of children. Provision is made for a range of circumstances (see Figure 1), which include providing support for children in need, investigating the circumstances of children thought to be at risk of significant harm and the accommodation or supervision of children. Children who are accommodated are said to be ‘looked-after’ and the local authority is able to ‘override’ parental responsibility for the child. There are two main legal routes to becoming looked-after: out-of-court arrangements under section 20, and the granting by a court of a care order under section 31 [4]. It is these court cases, referred to as public family law proceedings, which are the scope of this data resource profile.

Public family law proceedings are typically characterised by local authority applications for court orders to either receive a child into care or place the child under local authority supervision, in situations where the child is suffering, or likely to suffer from (i.e. at risk of) significant harm. In all such proceedings, a ‘Children's Guardian’ (a specialist social worker) from Cafcass is appointed to ensure that decisions made by the courts are in the child’s best interests. Although Cafcass is sponsored by the Ministry of Justice, it operates independently and provides information, advice and support for children and their families [5]. Cafcass guardians are qualified social workers and their role includes checking the local authority’s assessment and care plan, attending hearings, and advocating for the children they work with.

For most children, becoming looked-after and entering OOHC indicates experience or risk of maltreatment [4]. Types of maltreatment are physical, sexual, emotional abuse and neglect. These experiences can result in serious injury, disability and death, and has been linked to an increased risk of adverse outcomes later on in life, such as poor mental health, substance misuse and risky sexual behaviour [6,7]. In England, it is estimated that around 1 in 30 children will spend some time in OOHC before their 18th birthday, with evidence that this figure is rising [1]. Therefore, child maltreatment is a growing public health issue [8].

The role of the courts in public family law proceedings is to rule on applications made by local authorities to intervene in family life in order to safeguard child welfare. These rulings, or orders, grant certain permissions to the local authority, based upon the risk of significant harm; this can include making changes to a child's living arrangements, deciding upon the level of contact between children and parents, and deciding whether there need to be any changes to who has parental responsibility for a child. The most common public family law orders made concerning children are care orders, which place children under the care of their local authority (typically into OOHC) [9]. Other orders include: emergency protection orders, which allow the local authority to assume immediate parental responsibility for a child for up to eight days; supervision orders, where the court instructs the local authority to ‘advise, assist and befriend’ a child; and placement orders, which allow a child to be placed for adoption.

Cafcass also has a role to play in private family law proceedings, which typically concern disputes between private parties (such as between parents on relationship breakdown) about the upbringing of a child. Cafcass carries out initial safeguarding checks in such cases and may become more involved in a case where welfare concerns are identified. This data resource profile, however, will focus only on public family law case records (information on the private records is available elsewhere [10]). This profile also only covers England, similar law and systems are extant in the devolved countries of the United Kingdom but data collection is separate.

Data for research exists from 1 April 2007 onwards. Between 1 April 2007 and 31 March 2019, Cafcass has captured information on approximately 172,100 public family law cases, involving 282,300 children, and 349,600 adults (of which 289,300 are recorded as birth parents). Figure 2a shows how these numbers have increased steeply over time, while Figure 2b displays the considerable variation in the number of children subject to court proceedings for each local authority in 2017/18, proportional to their general child population. With points falling outside of the 99% confidence limit, this is evidence that many local authorities differ from the national average, as this level of variation is unlikely to be due to chance.

**Figure 2: Number of parents and children entering public law proceedings over time and local authority variation in the rate of child entry into public law proceedings.**
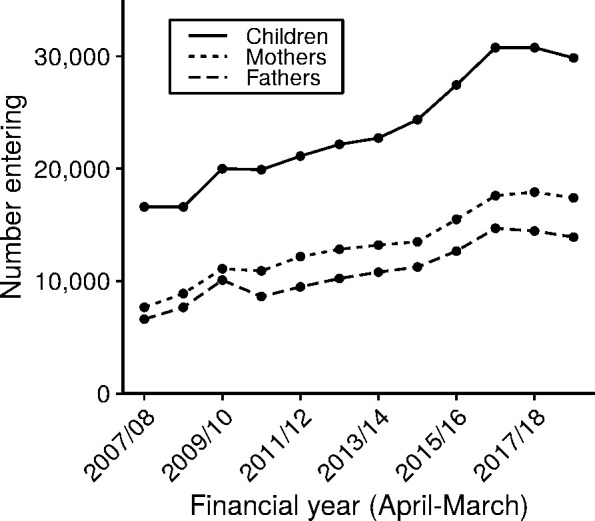

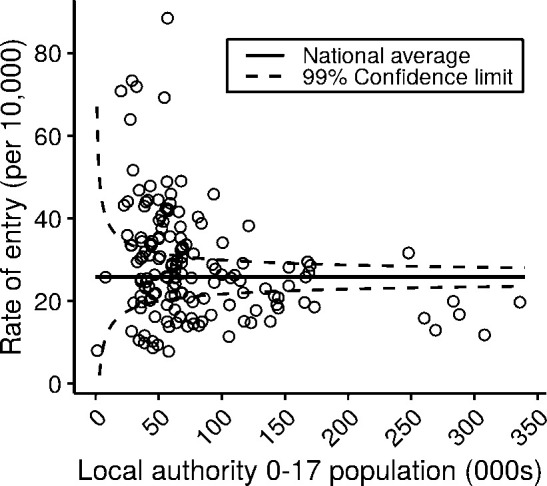
(a) Number of children, mothers and fathers entering public law proceedings, by financial year (April-March).

(b) Funnel plot of the rate of entry for children per 10,000 of the 0-17 local authority population in 2017/18.

## Data production

Cafcass maintains an administrative digital system to securely store case management records. This system supports more effective management of caseloads to improve workflow, as well as providing a monitoring function of the service it provides. Basic administrative information is recorded for each case that Cafcass guardians are involved in.

Much of the information that Cafcass collects comes directly from a standardised form sent to them directly by the applicant local authority. This form is called the C110A and covers applications for care orders, supervision orders or emergency protection orders [10,11]. On receipt of an application form, Cafcass creates a new case record within its system and assign a guardian who contemporaneously updates the case record. As part of their recording practice, on case closure, the guardian should attempt to supplement any additional information that was previously missed. The quality of case records is monitored and assured by a service manager.

In addition to a Cafcass guardian, children subject to public family law proceedings are usually also represented by a publicly-funded legal representative (known as the ‘tandem-model’ of representation) [12,13]. In the face of increasing demand for their services, Cafcass began to operate a model of “proportionate working” in 2010 [5]. This means directing resources in each case to activity that is essential. In some cases, the Guardian no longer attends all hearings. As a result, Cafcass no longer records interim legal outcomes, though final legal outcomes are still captured. 

## Data contents

### Structure of the data extract

**Figure 3: The timelines of a random sample of 100 public law cases based on the Cafcass data extract.**
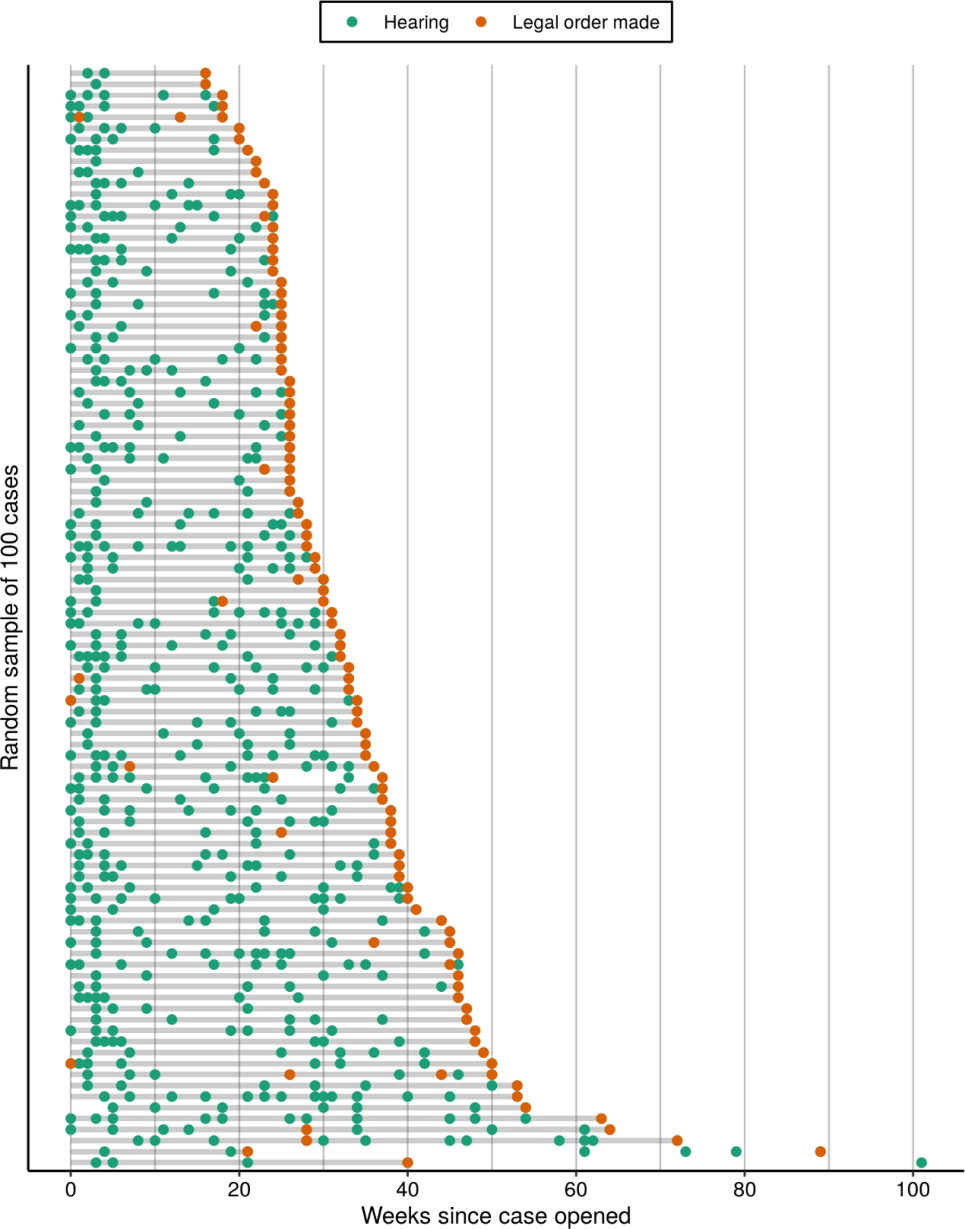
Timelines start when a case is first opened, and they show the length of the s31 care application (grey line), along with hearings (green) and legal orders being made (orange). Note that a case may not start with a care application, but often closes at the same time the application ends.

Cafcass records every application brought forward by a local authority, with a case being made up of one or more applications. Parents or caregivers are identified as parties to the application, with one or more children being subject. Other persons, such as non-subject siblings, non-party grandparents or a parent’s partner, can also be named on the application if relevant, though this is not common. The start date of an application is the date the local authority submitted the application to court, with the end date being the date for which a final legal order was made; from these two dates, an accurate measure of how long proceedings have lasted can be derived. Figure 3 displays a random sample of 100 cases from the data extract, demonstrating the variable length and complexity (with regard to sequences of hearings and legal orders) of these court proceedings.

In 2007, Cafcass began to use an electronic case management system, which was replaced in July 2014 with a newer system. These systems have captured data that, though not originally intended for research purposes, can be used to gain insights about family law proceedings, after data cleaning and preparation for research. What we describe below are tables which have derived from these two case management databases, by harmonising the differences between them to produce a set of data tables suitable for research (see Supplementary Appendix 1: Figure A1 for further detail).

### Measures collected

Within its case management system, Cafcass captures a large amount of information on adults, children, cases and the court process in public law. As summarised in Table 1, the pseudonymised data extract includes key measures from the case management system and these can be grouped into case and application characteristics, outcomes of applications for children and characteristics of the families involved. Generally, recording of these measures has improved over time, though the proportion of legal outputs recorded has decreased in recent years (see Supplementary Appendix 1: Table A1 for more details on missing values in key variables between 1st April 2007 and 31st March 2019).

**Table 1: Measures and characteristics available in the pseudonymised Cafcass data extract.** 

Case and application characteristics	Individual and family member characteristics	Outcomes of application
For all public family law proceedings:	For all people named in application:	For all children on cases:
Case and application ID Application type (e.g. emergency protection order, care order, supervision order, placement order) Court ID Local Authority ID Date application was made and date ended Date record was closed by Cafcass Dates of hearings attended by Cafcass Guardian	Person ID Week of birth Gender Ethnicity Lower-layer Super Output Area ID of the address postcode Religion First language Interpreter required (y/n) Disability (y/n) Access support required (y/n) Role in application (subject, party, other) Relationship with other named persons (e.g. child, mother, father, grandparent)	Legal output ID Legal output type (e.g. emergency protection order, care order, supervision order, placement order, order of no order) Order type (final / interim) Date order made

#### Case and application characteristics

Each case is given an internal, unique ID in Cafcass, as is each application. Cases are identified as private or public law, with the type of application(s) also being specified (e.g. application for care order, supervision order etc.). The local authority that submitted the application is recorded at the case level, with dates being recorded at the application level. Additionally, dates of hearings attended by guardians are recorded separately, as well as requested reports and their due dates.

#### Individual and family characteristics

**Figure 4: Children’s ages and family compositions in public family law proceedings starting in 2017/18.**
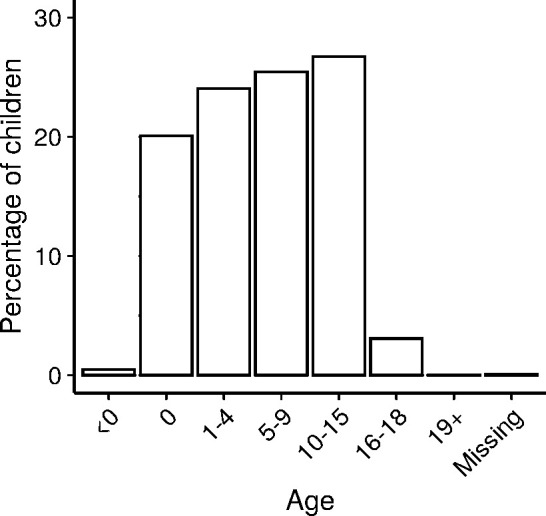

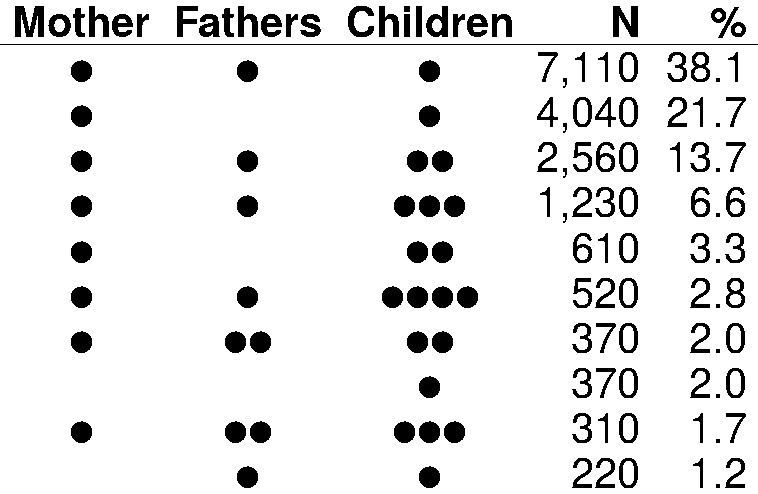
(a) Child age at the start of proceedings

Demographic information is recorded for each person named on an application (parents or carers who are party to proceedings, and children subject to them). For example, from Figure 4a, we can see that 44% of children assigned a Cafcass guardian in 2017/18 are aged between 0 and 4 years old, and 52% between 5 and 15 years old.

All details are expected to be kept up-to-date while the case is ongoing which means that, for some people, information on previous names and addresses is available, which have the potential to be used for linkage. Any assessments undertaken during proceedings (such as medical or psychiatric examination of the child) are generally stored as documents separate from the administrative system and are not available within this data extract.

Cafcass also records the relationships between the adults and children on an application, allowing researchers to identify families. Figure 4b summarises the most common family compositions recorded in 2017/18.

(b) Top 10 most frequent family compositions in proceedings.

#### Outcomes of application

**Figure 5: Legal outcomes for children in public family law proceedings in 2017/18 and how they relate with the age of the child.**
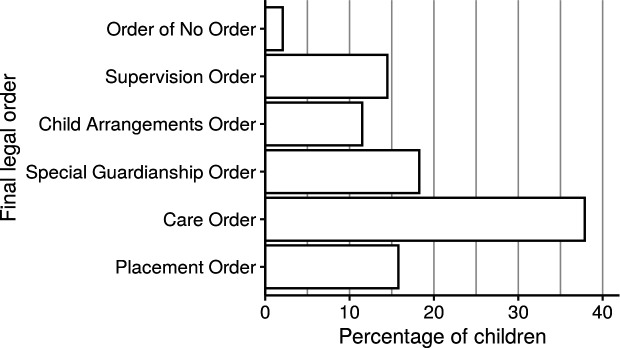

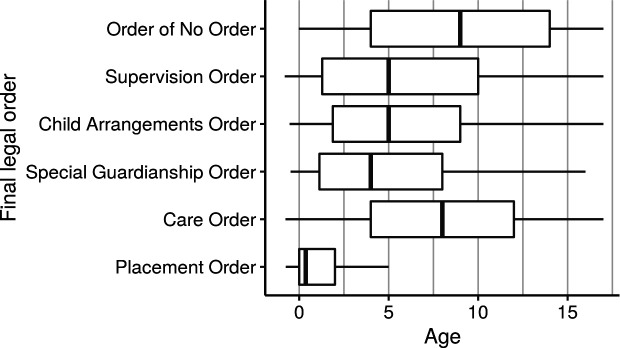
(a) Percentage of children receiving specific final legal orders in 2017/18.

Legal outputs can be made both during an application (such as an Interim Care Order) and when a case concludes (referred to here as the final legal output). More recently, Cafcass has reduced the recording of interim legal outputs as final legal output recording has taken priority. Examples of final legal outputs include: Order of No Order (ONO), Supervision Order (SO; the child remains in the family home), Child Arrangement Order (CAO; to regulate parental contact and living arrangements), Special Guardianship Order (SGO), Care Order (CO; the child is placed with foster carers), or Placement Order (PO; the child is placed with prospective adopters). Figure 5a displays how many children in the data extract are subject to these different final legal orders in 2017/18, whereas Figure 5b shows the distribution of their ages, by legal order. For example, while care orders are the most common legal order recorded overall, placement orders are more common among infants and young children.

(b) Distribution of age among children receiving specific final legal orders in 2017/18.

## Existing and ongoing work

### Risk of mothers and fathers returning to court

As Cafcass records parent-child relationships in the majority of cases, this enables researchers to link multiple public family law applications from the same individual or family group to identify returns to court over time. This was utilised in a pioneering study to quantify recurrence among mothers subject to care proceedings [14]. Their findings provided the first estimate of the risk of mothers re-entering care proceedings and suggested that many mothers subject to recurrent care proceedings are caught in a cycle of successive pregnancies with early intervention from children’s services. This work has been further developed by a study of fathers, vis-à-vis mothers, in recurrent care proceedings which has shown rates of recurrence as being 13% and 22% over five years, for fathers and mothers, respectively [15].

### Newborns in care proceedings

Due to the accurate and consistent recording of child demographics, research into sub-infant age groups has shown that while children aged less than one year constituted 27% of all children in care proceedings from 2007/08 to 2016/17, 36% of which were less than one week old [16]. Expressed as a rate of per 10,000 live births, overall there has been an increase from 15 to 35 per 10,000. Given the unique vulnerability of newborns, preventative action is understandable, however compulsory removal should be seen as a severe form of intervention in family life. Further work is needed to evaluate outcomes for this particular sub-population of children, both in childhood and later on in life. For example, on the implications of being placed into care so early on in life on early childhood development, mental and physical health, and education wellbeing.

### Linkage to other administrative datasets

Cafcass holds person identifiers (name, address at time of care proceeding and date of birth) on the adults and children that can be used to link the Cafcass data extract to other individual-level administrative databases. While linkage to other datasets is currently in its infancy there are a couple of notable projects: the Ministry of Justice has linked data collected by Cafcass to other family courts data as part of their Children in Family Justice Data Share (CFJDS) project, though the accuracy of this linkage is not currently known [17,18]. Additionally, Cafcass data is also being linked to administrative healthcare records in England, enabling research into the healthcare needs of mothers involved in care proceedings to inform interventions to safeguard vulnerable mothers and their children [19]. Within this project, two record linkages are being performed. The first is between Cafcass and data from the Clinical Record Interactive Search (CRIS) database at South London and Maudsley NHS Foundation Trust, which contains information on mental health service use in South London [20]. The second data linkage is between Cafcass and data from Hospital Episode Statistics [21], which contains patient-level information from admissions, outpatient appointments and A&E attendances to NHS hospitals in England. 

### Other work

The Nuffield Family Justice Observatory was created in March 2019 with the following aims: build capability in the use of administrative data for the Family Justice System, improve the supply of empirical evidence, and work collaboratively with stakeholders to identify priorities for new research or synthesis [22]. A major step towards that goal is making the data described here and that of Cafcass Cymru available to researchers, which is discussed in more detail in the Access section. Additionally, Cafcass England maintains a list of internal and external projects conducted using their data. For the full list, please refer to their website [23].

## Strengths and limitations

### Strengths

The Cafcass data extract provides national coverage of children involved in public family law proceedings in England and contemporaneous recording of case details, therefore mitigating participant selection and recall biases encountered with other types of study design. The longitudinal nature of this resource, coupled with the ability to identify parent-child relationships, presents the opportunity to follow individuals subject to public family law proceedings over time in order to monitor returns to court. As well as identifying birth parents, Cafcass often identifies the relationships between children and extended family members or non-biological carers giving new insights into the family structures of children involved in public family law proceedings (Figure 3b). Outside of consent-based cohort studies, there are few data sources where a researcher could accurately identify parent-child relationships and identifying parents via household address may perform poorly among transient groups and populations with high prevalence of family breakdown [24,25].

This resource allows researchers to study court-related and population-related trends over time that can be stratified by subgroups such as age, order type and length of proceedings, due to the abundance of information captured by Cafcass. In particular, it may also be possible to evaluate the impact of local interventions targeted at individuals and families subject to public family law proceedings, by comparing outcomes captured by Cafcass between geographical areas.

Further, the Cafcass data extract has great potential to be linked to other administrative databases, covering areas such as health, criminal justice, or education, to look at a broad range of outcomes among families, parents and children subject to public family law proceedings (for example, Cafcass data has already been linked to other family court data within the Ministry of Justice CFJDS project [17]). Any data linkage would be subject to the appropriate permissions being granted. 

### Limitations

A limiting factor of this resource arises from the nature of Cafcass’ work: Cafcass only collects data on cases in which they have been involved. Therefore, the data extract does not include out-of-court arrangements between local authorities and parents such as accommodating children under section 20 (Children Act 1989) — although these data are available in the CLA database [3].

Another caveat is data quality. Prior to April 2010, recording of information for cases was generally poor: less information was recorded about legal orders made and adults listed in a care application were assumed to be respondents (the child’s carers, most often the mother and father) in the case. However, missing data in case records has largely improved over time, as shown in Table A1. In addition, trends over time may reflect changes in recording and/or practice. Similarly, they may reflect changes to children’s services practice, early intervention provision, or availability other public services (e.g. housing, universal credit, public health programmes) that may indirectly affect demand for public family law intervention.

Due to the Cafcass policy of “proportionate working”, they moved to a model of only recording legal orders sufficient for the case to be closed i.e. those that are final. Additionally, no information is recorded on hearings in which a Cafcass Guardian did not attend. Additionally, case records are recorded contemporaneously and, while this reduces recall bias, records are not updated after involvement of the Cafcass Guardian ends. Because of this, some individuals have been erroneously assigned more than one unique ID in the case management system if they changed their last name and/or address on return to court – this can lead to an underestimation of recurrence in public family law proceedings.

Lastly, whilst one of the aims of the Nuffield Family Justice Observatory is to increase usage of available admin data, there is currently no linkage to other datasets that can be made available to researchers, though this is something that is being worked towards.

## Access

From January 2020, access to the Cafcass research data extract will be made available via the Secure Anonymised Information Linkage Databank (SAIL) [26]. Cafcass aims to transfer a refresh of the extract on a bi-annually basis. The SAIL Databank provides secure storage of anonymised individual-level administrative data for research. The anonymization process that the Cafcass data goes through means that all direct identifiers are removed (e.g. name, exact date of birth, address lines) and a unique anonymised person identifier is assigned, with a project-specific encrypted version of this ID provided uniquely to each project.

Subject to ethical approval and data sharing agreements, SAIL can also link datasets that it holds to one another, or to external data [27,28]. However, opportunities for data linkage are currently limited by the datasets held in SAIL; all other individual-level datasets currently in SAIL include data from the Welsh population only. Applications for data through SAIL follow a two-stage application. Firstly, researchers must discuss their project with a SAIL Databank analyst who will aide them in completing an initial application. Next, researchers must apply for approval from SAIL’s Information Governance Review Panel. Cafcass also has a final say on all application to SAIL for this extract.

The associated cost of a project wanting to use Cafcass data within the SAIL platform is dependent on the unique requirements, resources and support required by each project. However, the general costing model is available from SAIL upon request.

If successful, applicants must undergo Safe Researcher training; a list of suitable courses can be found on their website. Data is accessed via the SAIL Gateway which includes SQL querying tools and applications for analysis such as Microsoft Office, SPSS and R (Stata is available for a small licensing fee). Further information is available from the SAIL website [29]. Access arrangements are subject to the on-going relationship between Cafcass and SAIL and any future developments.

Regarding linkage to other datasets, there exists a tripartite agreement between SAIL, Cafcass and NHS Wales Informatics Service (NWIS), in which NWIS acts as a Trusted Third Party to perform the linkage and assignment of the unique person identifier to SAIL to be combined with the non-identifiable data through the standard split file process [30]. This ensures that data linkages can happen while protecting the identities of those in the data.

## Public involvement

Cafcass makes publically available statistics regarding the public and private family law cases they are involved in, as well as listing research projects carried by themselves and external researchers [23]. While Cafcass engages with children and families to inform policy and practice, they do not currently involve members of the public in the approvals process for external research projects using the data described here [31]. However, the SAIL Databank operate a Consumer Panel made up of members of the public which then advise on the development of ideas and the approval of bids as part of their Information Governance Review Panel, as well as on the dissemination of research findings [32].

We encourage all those interested in using this data resource to engage with members of the public regarding the development, interpretation, and dissemination of research. Individuals with experience of public family law proceedings are a seldom heard from group and, therefore, research using Cafcass data presents an opportunity to engage with members of this population to improve their representation in public involvement activities. Involving members of the public in research, particularly members of the population under study, can help to preserve the focus of research objectives to the study population, ensure any findings are relevant and important to the study population, and improve the ability for research to positively impact policy and practice. 

## Acknowledgments

We would like to thank the Children and Family Court Advisory and Support Service for collecting and allowing researchers to access their data and, in particular, Richard Green for reviewing this publication for accuracy in the description of their work and data access. We would also like to thank Lucy Griffiths and Ashley Akbari at SAIL Databank for reviewing the content relating to SAIL and its processes, and Bachar Alrouh for his contributions towards making the data available in SAIL. This work was funded by the Nuffield Foundation (grant number: KID/42838) as well as the NIHR GOSH BRC. The views expressed are those of the author(s) and not necessarily those of Cafcass, the Nuffield Foundation, the NHS, the NIHR or the Department of Health.

## Contributors

SB had approved access to the source Cafcass records and carried out all data manipulation and production of the data extract, as well as the descriptive results presented here. RP led on writing this paper. All authors inputted to the work carried out and have critically reviewed the contents of the manuscript and consent to its publication.

### Ethics

Ethical approval was obtained from the UCL Research Ethics Committee (application number 10803/001), the Lancaster University FAS-LUMS Research Ethics Committee (application number FL16179), and the Cafcass Research Governance Committee to work with the Cafcass data.

## Supplementary Material

Supplementary Appendix 1
