# Supplementary material for: Data Resource: Children and Family Court Advisory and Support Service (Cafcass) public family law administrative records in England
Source: Int J Popul Data Sci. 2020 Mar 26;5(1):1159. doi: 10.23889/ijpds.v5i1.1159 (PMC7482375; doi:10.23889/ijpds.v5i1.1159)
Supplement: Supplementary Appendix 1 [file ijpds-05-1159-s001.pdf]

Data Resource: Children and Family Court Advisory and Support Service (Cafcass) public family law administrative records in England

Bedston SJ et al. IJPDS

Supplementary Appendix 1: Additional tables and figures

Figure A1 and Table A1 may be useful for a researcher who is considering using the Cafcass data extract for their own purposes. Figure A1 shows the structure of the tables available in the extract, while Table A1 reports on the % missing within each column and relation (e.g. link between the 'person' and 'person address' tables). The 'application person' table was split in two, adult and child, for the purposes of reporting on % missing as there is a distinct difference in the patterns, and only children are made subject to legal orders.

Figure A1: Relationships between key tables available in the Cafcass data extract, listing the variables available in each. All date variables in the event extract are rounded down to the previous Monday (DFJ = Designated Family Judge; PR = parental responsibility; LSOA = Lower-layer Super Output Area).

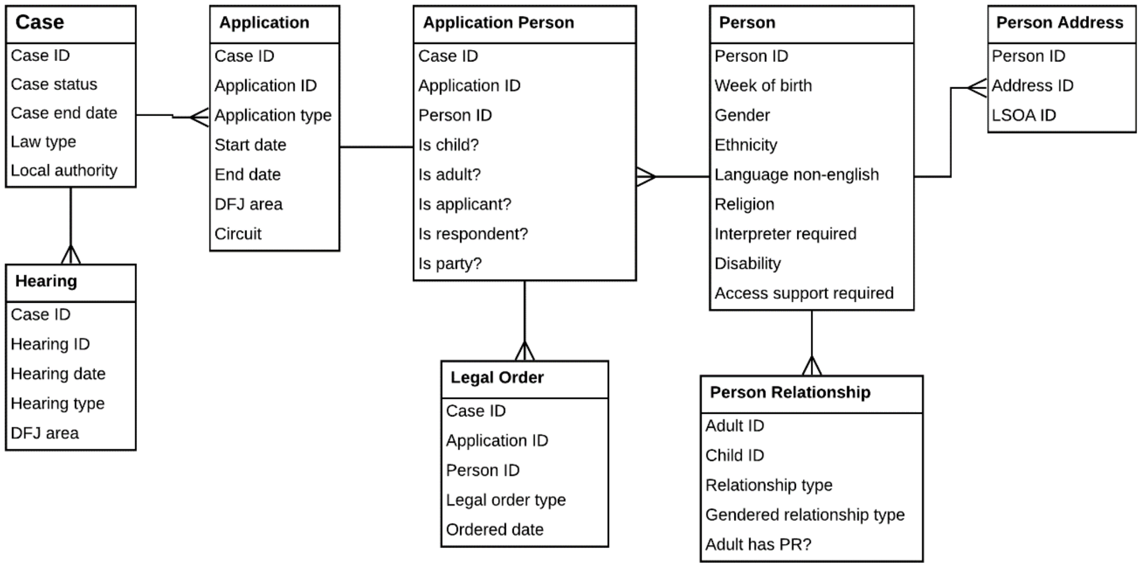

Table A1: Percent missing among key variables captured in Cafcass case records, by financial year (April-March), from 2007/08 to 2018/19.

**Table**	**Variable**	**'08**	**'09**	**'10**	**'11**	**'12**	**'13**	**'14**	**'15**	**'16**	**'17**	**'18**	**'19**
Case (N)		10,400	10,400	12,100	11,800	12,900	13,500	13,800	14,700	16,800	18,800	18,700	18,200
	Status	0.0	0.0	0.0	0.0	0.0	0.0	0.0	0.0	0.0	0.0	0.0	0.0
	Application	0.0	0.0	0.0	0.0	0.0	0.0	0.0	0.0	0.0	0.0	0.0	0.0
	Local authority	0.7	0.7	0.8	0.9	0.2	0.1	0.1	1.0	1.0	0.6	0.5	0.8
	Hearing	0.1	0.2	0.5	0.6	0.1	0.0	0.1	1.1	1.3	1.1	1.4	1.3
	Legal output	32.8	0.0	0.0	0.0	15.8	15.1	0.0	0.0	0.0	0.0	0.0	0.0
	Child subject	0.0	0.0	0.0	0.0	0.0	0.0	0.1	0.2	0.2	0.3	0.3	0.3
	Adult party	1.3	0.9	0.6	0.5	0.4	0.3	0.3	0.6	0.6	0.6	0.5	0.6
	Mother party	2.7	2.3	2.1	2.5	2.3	1.8	1.8	2.3	2.1	2.2	1.8	2.2
	Father party	17.3	16.3	14.1	22.3	22.9	21.8	20.6	20.7	21.6	21.0	22.8	24.2
Application (N)		12,800	12,800	15,200	15,300	16,900	18,100	18,400	19,100	21,800	24,300	25,100	24,500
	Start date	0.0	0.0	0.0	0.0	0.0	0.0	0.0	0.0	0.0	0.0	0.0	0.0
	Application type	0.0	0.0	0.0	0.0	0.0	0.0	0.0	0.0	0.0	0.0	0.0	0.0
	DFJ area	1.9	1.8	1.2	1.0	1.0	0.7	0.9	0.6	1.0	1.2	2.1	3.3
	Child subject	0.1	0.1	0.0	0.0	0.0	0.0	0.4	1.4	1.3	1.5	1.0	0.8
	Adult party	1.2	1.4	3.3	2.1	1.4	1.1	1.1	2.3	2.0	1.8	1.2	1.1
	Mother party	2.5	2.8	4.9	4.2	3.2	2.6	2.6	4.0	3.5	3.3	2.5	2.7
	Father party	17.0	16.8	16.9	24.7	25.0	24.5	22.7	23.5	24.3	23.1	25.1	25.6
	End date	0.0	0.0	0.0	0.0	0.0	0.0	0.4	1.5	1.6	1.8	4.1	42.6
Application child (N)		19,800	19,800	24,200	24,400	26,100	28,200	28,500	29,600	33,300	37,100	38,600	37,800
	Week of birth	0.0	0.0	0.0	0.1	0.1	0.0	0.0	0.1	0.1	0.1	0.1	0.0
	Gender	0.0	0.0	0.0	0.0	0.0	0.0	0.0	0.0	0.0	0.0	0.0	0.0
	Ethnicity	28.2	32.8	55.7	60.8	62.3	71.1	81.3	75.0	24.0	8.8	4.3	10.7
	Language	31.4	36.5	58.8	62.6	63.3	71.8	81.4	75.1	24.1	9.7	4.4	9.9
	Relationship	23.2	10.4	4.9	4.4	2.1	1.8	1.4	5.8	5.2	3.2	1.2	1.9
	LSOA ID	46.8	43.9	42.7	40.1	41.7	40.6	39.0	42.6	43.7	40.3	36.5	35.8

Legal output	0.3	0.3	0.1	0.1	0.4	1.0	2.9	0.1	0.4	0.3	3.5	45.3
Application adult (N)	27,900	28,300	32,400	29,100	32,200	34,800	35,600	36,800	41,900	47,100	47,900	46,500
Week of birth	27.2	23.7	21.8	15.4	14.1	12.6	11.2	10.8	10.3	9.5	8.7	7.8
Gender	0.0	0.0	0.1	0.1	0.1	0.1	0.1	0.1	0.1	0.1	0.0	0.1
Ethnicity	40.9	43.9	60.7	61.0	62.4	67.6	74.8	68.9	26.4	12.3	6.7	11.9
Language	41.7	45.4	61.8	61.8	62.6	67.6	73.9	68.2	24.2	10.0	4.6	9.3
Relationship	32.7	23.8	17.2	12.6	10.8	10.7	10.0	10.7	8.6	6.4	3.8	3.9
LOSA ID	24.0	17.6	16.1	14.0	13.2	12.6	11.5	13.2	13.8	13.8	12.2	12.9
Hearing (N)	56,600	67,500	96,300	100,000	98,000	90,200	70,400	65,900	76,600	88,800	93,700	80,800
DFJ area	0.5	0.3	0.2	0.1	0.2	0.1	0.2	0.1	0.1	0.1	0.1	0.2
Hearing date	0.0	0.0	0.0	0.0	0.0	0.0	0.0	0.0	0.0	0.0	0.0	0.0
Legal output (N)	54,500	52,600	64,600	73,700	75,700	64,300	48,900	36,600	41,600	48,100	48,200	26,300
Output type	52.6	39.4	10.5	0.0	0.0	0.0	0.0	0.0	0.0	0.0	0.0	0.0
Ordered date	0.0	0.0	0.0	0.0	0.0	0.0	0.0	0.0	0.0	0.0	0.0	0.0
